# Perceived E-Health Literacy and Cyberchondria Among Undergraduate Sports Science Students: A Cross-Sectional Study

**DOI:** 10.3390/healthcare14172862

**Published:** 2026-09-05

**Authors:** Cemile Nihal Yurtseven, Mert Canbaz

**Affiliations:** 1 Department of Sports Management, Faculty of Sports Sciences, Istanbul University-Cerrahpasa, 34320 Istanbul, Turkey; nihalyurtseven@iuc.edu.tr; 2 Department of Anesthesiology and Reanimation, Istanbul Faculty of Medicine, Istanbul University, 34093 Istanbul, Turkey; 3 Department of Biostatistics, Istanbul Faculty of Medicine, Istanbul University, 34093 Istanbul, Turkey

**Keywords:** e-health literacy, cyberchondria, digital health behavior, online health information seeking, health anxiety, student well-being, university students, sports science students

## Abstract

**Objectives:** The rapid digitalization of health communication has made electronic health (e-health) literacy an essential competency, while also increasing concerns about cyberchondria. This descriptive–correlational study examined perceived e-health literacy and cyberchondria among students enrolled in a Faculty of Sports Sciences and evaluated their associations with selected socio-demographic and behavioral factors. **Methods:** Data were collected between February and May 2025 using a Socio-Demographic Information Form, the e-Health Literacy Scale (eHEALS), and the Short-Form Cyberchondria Severity Scale (CSS-12). Quantitative data were analyzed using group comparison tests, correlation analyses, effect size estimates, and multivariable linear regression. **Results:** Students demonstrated eHEALS scores close to the upper end of the possible range, together with considerable cyberchondria severity scores. Cyberchondria severity was positively associated with perceived e-health literacy; this association remained statistically significant but weak after adjustment for daily internet use, gender, age, academic year, and department. Significant differences were also observed according to daily internet use, gender, academic year, and department. **Conclusions:** These findings highlight the dual role of digital health platforms among students engaged in health- and performance-related fields. Educational interventions should promote critical appraisal of online health information and psychologically safe digital health behaviors.

## 1. Introduction

According to recent evidence, university student cohorts form a significant risk group prone to psychosocial health difficulties and problematic health behaviors [1]. Furthermore, the expanding modern practice of utilizing online ecosystems to retrieve health data has introduced substantial risks closely tied to specific psychological distress factors. As a result, individuals often turn to internet-based medical searches as a dysfunctional strategy to manage their health-related fears, which is increasingly contributing to psychological distress among the student population [2]. This situation, especially when combined with health anxiety and excessive online health information searching, has exacerbated problems and led to cyberchondria [3].

In e-health literacy, digital technologies play a crucial role. Technological progress in the modern digital era has fundamentally transformed how individuals retrieve health data. Due to this heightened accessibility, the medical domain widely embraces the deployment of online platforms, mobile applications, and social media channels to advocate preventive health behaviors and facilitate care [4]. Consequently, many individuals, including young adults and university students, increasingly use digital sources to seek health-related information, often perceiving online channels as accessible and efficient alternatives to conventional information sources [5].

This concept represents an individual’s perceptive capacity to locate, comprehend, appraise, and critically use health information obtained via digital networks to resolve wellness challenges [6,7]. As digital communication tools expand and technological innovations accelerate, navigating healthcare frameworks in today’s data-driven society demands a high degree of e-health literacy, rendering it a vital skill for modern life [8]. According to the conceptual framework of Norman and Skinner, electronic health literacy represents a multifaceted synergy of cognitive, social, and technical proficiencies. With the widespread use of smartphones and the Internet, individuals increasingly retrieve, evaluate, and use information from various digital health platforms, including social media, web search utilities, dedicated health portals, and mobile applications [6]. Nonetheless, evidence suggests that simply obtaining health information is insufficient for driving actual behavioral improvements [9]. E-health literacy may therefore influence how consumers filter credible information, understand complex medical content, and make informed health-related choices. Evidence suggests that a strong correlation exists between e-health literacy levels and enhanced wellness indicators, which subsequently fosters a more robust commitment to proactive, preventive health behaviors among individuals [10]. In this context, e-health literacy may contribute to self-management, shared decision-making, and the ability to distinguish evidence-based information from misinformation [11]. Consequently, higher e-health literacy may support individuals’ ability to critically appraise online health information and recognize potentially misleading or unreliable content [12].

E-health literacy is a compound construct, as it comprises a variety of digital health competence components. These varying components can enable an individual to engage in positive health behavior. However, they can also be detrimental if not properly balanced, as previously identified [13,14]. Positive effects of higher e-health literacy can include greater awareness of the need to critically evaluate online information about the topic of interest or an increase in knowledge about health issues. Nonetheless, negative effects can include information overload, health anxiety and/or cyberchondria. Hence, future scholarly inquiries must delve deeper into the scope of e-health literacy’s impact on behavioral outcomes. Concurrently, exploration is required regarding how this competence can be leveraged for positive affect regulation, all while sustaining a balanced, non-anxious curiosity toward disease concepts [11]. While advanced e-health literacy commonly functions as a primary driver of positive behavioral modifications linked to health cultivation and structured decision-making, it can paradoxically precipitate counterproductive outcomes. A prominent example includes the escalation of health-related anxieties triggered specifically by internet-based medical searches [15].

To effectively navigate and oversee their personal health, higher education students commonly turn toward various virtual platforms, reflecting a widespread reliance on digital technologies and the Internet for health-related data retrieval. However, reliance on multiple online sources may expose students to information overload, health anxiety, ineffective self-management, and exacerbation of health-related concerns. Therefore, students need skills for searching, evaluating the credibility of information, and managing online health content responsibly. Evidence indicates that effective online health information-seeking pathways are significantly driven by individuals’ traits and state e-health literacy. To foster positive public health outcomes, it is therefore crucial to design interventions aimed at elevating these specific literacies within student cohorts [16].

E-health literacy is a multidimensional construct that includes critical evaluation skills, digital communication competencies, and information management skills. These skills are needed to assess the appropriateness and reliability of health information within the digital health ecosystem. Specifically, e-health literacy requires educational approaches to foster critical thinking, digital competence, and responsible interaction rather than simply improving access to digital resources. Paige et al. suggested that these skills can be developed through contextualized and realistic training environments, learner-centered pedagogical activities, and tasks that promote advanced cognitive processing [17].

Focusing specifically on sports science students, this study addresses a population whose academic training is closely related to physical activity, injury prevention, rehabilitation, nutrition, health promotion, and performance-related topics. Previous studies have examined the relationship between perceived e-health literacy and cyberchondria among undergraduate students and health sciences students using similar scale-based approaches [13,18,19]. However, evidence regarding undergraduate sports science students remains comparatively limited. Therefore, the present study does not claim to introduce a new theoretical mechanism; rather, it evaluates this previously described relationship within a specific educational context and examines whether the association remains after adjustment for demographic and educational characteristics. Especially after the COVID pandemic, with the increased levels of health anxiety, there has been a rise in reliance on online platforms for health information, resulting in problems with accessing inaccurate or inappropriate health information. The current inquiry tests two primary hypotheses:

**H1.**  *Cyberchondria severity shares a significant positive relationship with e-health literacy capacities within the sports science student cohort.*

**H2.**  *The extent of daily internet exposure significantly correlates with both digital health literacy scores and levels of cyberchondria.*

Operationally, cyberchondria refers to the pathological and irrational surge in online health monitoring and search practices, triggered by an exaggerated, anxious preoccupation with potential bodily symptoms.

The reality is that the intense search for general information about health and well-being can lead to situations of anxiety and/or distress, resulting in impairing mental well-being. Accordingly, this study aimed to investigate the relationship between perceived e-health literacy and cyberchondria severity in a cohort of sports science students, as well as how these scores differed according to the assessed demographic, educational, and behavioral variables, including age, gender, department, academic year, and daily internet use. Students in the health and sports sciences sector have access to advice on physical activity, injury prevention, rehabilitation, nutrition, performance enhancement, health promotion, and much more. These considerations suggest that when higher education cohorts specializing in sports sciences report higher perceived e-health literacy, they may feel more confident in accessing and using electronic health information; however, this perceived ability should be supported by critical appraisal, source verification, and emotional self-regulation skills.

## 2. Materials and Methods

### 2.1. Study Design and Participants

This descriptive–correlational, cross-sectional study was conducted among undergraduate students enrolled in the Faculty of Sports Sciences at a public higher education institution during the 2024–2025 academic year. The study aimed to examine the association between e-health literacy and cyberchondria levels in this student population.

A census approach was used, and all eligible students enrolled in the faculty were invited to participate. The target population consisted of 480 students. Of these, 438 students participated in the study, corresponding to a participation rate of 91.2%. Twenty-six participants were excluded because of incomplete questionnaire data. Because these incomplete forms did not consistently contain sufficient demographic and scale-related information, a formal comparison between included and excluded participants could not be performed. A complete-case analysis was therefore used, and the final analysis included valid responses from 412 students, representing 85.8% of the target population.

Students who were enrolled in the Faculty of Sports Sciences during the study period and agreed to participate were included. Participants with incomplete responses in the data collection forms were excluded from the analysis. Data were collected using a self-administered online questionnaire. All eligible students were invited to participate through faculty-based communication channels and course-related announcements. The invitation included brief information about the study purpose, voluntary participation, confidentiality, and informed consent. Students who agreed to participate completed the questionnaire electronically. Study data were collected between February and May 2025 using a Socio-Demographic Information Form, the e-Health Literacy Scale (eHEALS), and the Short-Form Cyberchondria Severity Scale (CSS-12).

### 2.2. Ethical Approval and Consent

This study was conducted in accordance with the Declaration of Helsinki. Ethical approval was granted by the Istanbul University-Cerrahpaşa Social Research Ethics Committee, Istanbul, Turkey (Approval No: 2025/67). Participation was voluntary, and informed consent was obtained from all participants before data collection.

### 2.3. Data Collection Instruments

#### 2.3.1. Socio-Demographic Information Form

The Socio-Demographic Information Form was used to collect information on participants’ demographic, educational, and behavioral characteristics, including age, gender, department, academic year, and daily internet use.

#### 2.3.2. e-Health Literacy Scale

Participants’ perceived e-health literacy was assessed using the Turkish version of eHEALS. The original scale was developed by Norman and Skinner, and its Turkish validity and reliability were established by Gencer [6,20]. The scale is a self-report instrument consisting of eight items that evaluate individuals’ perceived ability to find, understand, evaluate, and use health information obtained from electronic sources.

Each item is scored on a 5-point Likert scale ranging from 1 (“strongly disagree”) to 5 (“strongly agree”). The total score ranges from 8 to 40, with higher scores indicating higher perceived e-health literacy. In the present study, eHEALS total scores were summarized and analyzed as continuous scores. In the Turkish validation study, Cronbach’s alpha coefficient was reported as 0.86. In the present study, Cronbach’s alpha coefficient was 0.82.

#### 2.3.3. Short-Form Cyberchondria Severity Scale

Cyberchondria severity was assessed using the Turkish short form of CSS-12. The original scale was developed by McElroy et al., and its Turkish adaptation and validation were conducted by Yorgancıoglu-Tarcan et al. [21,22]. The scale consists of 12 items across four dimensions: excessiveness, distress, reassurance-seeking, and compulsion.

Items are rated on a 5-point Likert scale ranging from 1 (“never”) to 5 (“always”). The original CSS-12 score is interpreted on a 12–60 scale, with higher scores indicating greater cyberchondria severity. In the present study, CSS-12 was treated as a continuous score on the original 12–60 metric. For consistency with conventional CSS-12 total-score reporting, descriptive median and observed range values were presented as whole-number values. This presentation did not affect the inferential analyses or the interpretation of the findings. In the current study, Cronbach’s alpha coefficient was 0.87.

### 2.4. Statistical Analysis

Statistical analyses were performed using IBM SPSS Statistics version 27.0 (IBM Corp., Armonk, NY, USA) and R software version 4.5.1 (R Foundation for Statistical Computing, Vienna, Austria). Descriptive statistics were presented as mean ± standard deviation (SD), median, minimum–maximum values, frequencies, and percentages, as appropriate. The eHEALS and CSS-12 scores were analyzed as continuous variables. The normality of continuous variables was assessed using the Kolmogorov–Smirnov test. For group comparisons, variance homogeneity was assessed before selecting the appropriate test. Independent-samples *t*-tests or Welch’s *t*-tests were used for two-group analyses, whereas one-way ANOVA or Welch ANOVA was used for comparisons involving more than two groups, according to variance homogeneity. Effect sizes were reported as Cohen’s d for two-group comparisons and eta-squared (η^2^) for global multi-group comparisons, together with 95% confidence intervals. When global multi-group comparisons were statistically significant, post hoc pairwise comparisons were performed using Tukey HSD or pairwise Welch *t*-tests with Holm correction, as appropriate. Multiple testing was addressed by using adjusted post hoc procedures where applicable. Correlations were assessed using Pearson correlation for continuous variables and Spearman correlation for analyses involving academic year, which was treated as an ordinal variable. A multivariable linear regression model was constructed to evaluate whether perceived e-health literacy was independently associated with CSS-12 score after adjustment for daily internet use, gender, age, academic year, and department. Regression results were reported with unstandardized coefficients, 95% confidence intervals, standardized beta coefficients, *p*-values, variance inflation factors, adjusted R^2^, and diagnostic checks. Because heteroscedasticity was detected in the regression model, HC3 robust standard errors were used for confidence intervals and *p*-values. Statistical significance was set at *p* < 0.05.

## 3. Results

Table 1 delineates the descriptive statistics and baseline sociodemographic characteristics corresponding to the sampled higher education students pursuing sports science pathways. Of the 412 undergraduate students who successfully completed the data collection instruments, 53.4% identified as female, whereas 46.6% were male. The largest group was the “Sports Management” department (35.9%). Overall, more than a quarter of the participants (26.7%) were fourth-year students.

Table 2 outlines the descriptive statistics for the main continuous variables within the current study population. The mean score of the eHEALS was 37.1 ± 7.4 and the median was 40.0, with scores ranging from 8.0 to 40.0. Hence, the participants exhibited scores close to the upper end of the possible eHEALS range. The mean CSS-12 score was 31.8 ± 9.1, with a median of 32 and an observed range of 14 to 55. Regarding the age distribution, the participating sports science cohorts had a calculated mean of 21.4 years. The study found that the average daily internet use among participants was 4.9 ± 2.2 h/day (median: 4.9, range: 1.0 to 12.0 h/day).

Table 3 summarizes the comparisons of eHEALS and CSS-12 scores according to sociodemographic and educational variables. Female students had slightly higher eHEALS scores than male students, although this difference was close to the conventional significance threshold, while CSS-12 scores were significantly higher among female students. Daily internet use was significantly associated with both outcomes, with progressively higher eHEALS and CSS-12 scores observed across increasing internet-use categories. Significant differences were also observed across departments for both eHEALS and CSS-12 scores; Coaching Education students had the highest scores for both outcomes, whereas Sports Management students had the lowest CSS-12 scores. Academic year was significantly associated with both eHEALS and CSS-12 scores, with generally higher scores among students in more advanced academic years. Post hoc pairwise comparisons are presented in Supplementary Table S1; these analyses showed significant differences between daily internet-use groups for both outcomes, significant department-based differences particularly for CSS-12, and academic-year differences mainly between lower-year and upper-year students.

Table 4 presents the correlation analyses between academic year, daily internet use, perceived e-health literacy, and cyberchondria severity. Since academic year is an ordinal variable, Spearman correlation was used for analyses involving academic year, whereas Pearson correlation was used for associations between continuous variables. Academic year was not significantly correlated with daily internet use (rho = 0.02, 95% CI: −0.07 to 0.12, *p* = 0.647), but it showed significant positive correlations with both eHEALS score (rho = 0.47, 95% CI: 0.40 to 0.55, *p* < 0.001) and CSS-12 score (rho = 0.31, 95% CI: 0.22 to 0.40, *p* < 0.001). Daily internet use was moderately and positively correlated with both eHEALS score (r = 0.45, 95% CI: 0.37 to 0.52, *p* < 0.001) and CSS-12 score (r = 0.53, 95% CI: 0.45 to 0.59, *p* < 0.001). A significant positive correlation was also observed between eHEALS and CSS-12 scores (r = 0.54, 95% CI: 0.46 to 0.60, *p* < 0.001), indicating that higher perceived e-health literacy was associated with greater cyberchondria severity in the unadjusted correlation analysis.

Table 5 presents the multivariable linear regression model constructed to identify independent predictors of CSS-12 score. After adjustment for daily internet use, gender, age, academic year, and department, eHEALS score remained independently but weakly associated with CSS-12 score (B = 0.07, 95% CI: 0.00 to 0.14, standardized beta = 0.06, *p* = 0.041). This association was statistically significant but small in magnitude. In contrast, daily internet use, academic year, and department showed stronger standardized associations with CSS-12 score. Daily internet use was also independently associated with higher CSS-12 scores (B = 2.04, 95% CI: 1.81 to 2.28, standardized beta = 0.49, *p* < 0.001). Female gender, academic year, and department were significant independent predictors, whereas age was not significantly associated with CSS-12 score. Compared with students in Sports Management, students in Coaching Education and Physical Education Teaching had substantially higher CSS-12 scores. The overall model explained a large proportion of the variance in CSS-12 scores (adjusted R^2^ = 0.753). Diagnostic checks showed no relevant multicollinearity, with a maximum VIF of 1.27; because the Breusch–Pagan test indicated heteroscedasticity, HC3 robust standard errors were used for confidence intervals and *p*-values.

## 4. Discussion

The curriculum for sports science students includes subjects such as structural human movement, body kinematics, nutritional sciences, and athletic trauma. This specialized experience provides them with knowledge about strategies for physical conditioning, therapeutic recovery, functional performance, and overall well-being. While such engagement may be associated with higher perceived e-health literacy, the concomitant pressure of athletic injuries and performance-related concerns may also be related to repeated web-based symptom checking and cyberchondria-related behaviors. These interpretations should be considered cautiously, as injury history, athlete status, and performance anxiety were not directly measured in this study. In addition, symptom-searching behavior and individual-level curricular exposure were not assessed; therefore, these factors should be regarded as contextual considerations rather than tested explanatory variables or sport-specific mechanisms.

Digital health literacy functions as a cornerstone of modern public health, as it empowers populations to navigate, synthesize, and validate medical data encountered online. Conducted within the specific context of an Istanbul-based sports science faculty, this inquiry sought to decipher how perceived e-health literacy levels relate to the severity of cyberchondria among undergraduate students. The preferred method for coping with daily health problems is increasingly shaped by digital access and online health information seeking. Young men and women in the health and sports sciences sector have access to advice on physical activity, injury prevention, rehabilitation, nutrition, performance enhancement, health promotion, and many other topics. In the present study, students specializing in sports sciences had eHEALS scores close to the upper end of the possible scale range, indicating relatively elevated self-reported perceived e-health literacy rather than a categorically defined high level. The Emotional Burden area reflects mental distress and anxiety related to information obtained online. The psychological burden associated with managing e-health data appears to be significantly amplified among cohorts characterized by higher cyberchondria severity scores. The health problems arising from this impact are generally resolved through healthcare professionals. Our findings showed a positive association between perceived e-health literacy and cyberchondria severity, suggesting that greater confidence in accessing and using online health information may coexist with higher cyberchondria-related distress. Individuals with high levels of cyberchondria generally express mental distress and anxiety related to their health; this also indicates greater engagement with online health information rather than objectively verified digital competence [23,24]. However, it is thought that having information about health issues can increase psycho-emotional burden, but this burden should be managed with appropriate filtering mechanisms. Given that higher perceived e-health literacy may support more critical engagement with health-related information, developing strategic intervention protocols is essential to address cyberchondria-related risks associated with frequent or prolonged health-related internet use [17].

Despite eHEALS scores being close to the upper end of the possible range, participants were found to exhibit substantial cyberchondria severity scores simultaneously. This finding suggests that perceived e-health literacy alone does not protect individuals from health-related anxiety [25,26]. Individuals with high self-efficacy in digital health navigation tend to initiate web searches much more frequently. This frequent interaction with digital health information may be associated with greater exposure to contradictory, alarming, or inaccurate digital material [23,24]. Paradoxically, an overabundance of digital health resources may exacerbate user uncertainty instead of alleviating anxiety, serving as a critical catalyst for the escalation of cyberchondria-related behaviors.

A relevant but modest finding of this study is the significant positive correlation between perceived e-health literacy and cyberchondria severity (r = 0.54, 95% CI: 0.46 to 0.60, *p* < 0.001). Traditionally, e-health literacy has shown results consistent with other studies that have presented it as a protective factor enabling individuals to identify reliable sources of information and make informed health decisions [6]. However, our findings are consistent with evidence suggesting that frequent online health information seeking may increase exposure to anxiety-provoking content and contribute to cyberchondria-related behaviors [27]. In the multivariable regression model, perceived e-health literacy remained independently but weakly associated with CSS-12 score after adjustment for daily internet use, gender, age, academic year, and department. This indicates that the relationship between perceived e-health literacy and cyberchondria severity persisted even after accounting for key sociodemographic and educational factors, although its effect size was small compared with daily internet use, academic year, and department. Accordingly, perceived e-health literacy should be interpreted as a weak independent correlate rather than as the main determinant of cyberchondria severity in the adjusted model. This counterintuitive outcome implies that the utility of digital health literacy extends beyond mere technical retrieval proficiency; it fundamentally hinges upon a user’s capacity for objective verification and effective psycho-emotional self-regulation during exposure to medical data.

It was initially hypothesized that a significant positive linear relationship would emerge between the amount of time spent online daily and the participants’ digital health literacy scores. Aligning with the conceptual frameworks established by Barke et al. and Fergus and Spada, our findings indicated that daily internet use was positively associated with both perceived e-health literacy and cyberchondria severity [28,29]. It is also consistent with studies showing that individuals who engage in more online interactions will find online health information more easily and thus develop good e-health literacy in searching for health information. Although the Internet provides accessible and convenient health information, frequent reliance on online sources may reinforce repeated health information seeking and increase exposure to information overload, particularly among individuals with health-related concerns [30]. An excessive number of information sources may be associated with information overload or health anxiety, making it more difficult for individuals to evaluate the relevance and reliability of health-related information [28,29]. These outcomes closely align with the empirical insights of Vismara et al., who deduced that extensive internet exposure may be associated with maladaptive cognitive and behavioral traits [27]. However, academic year was not significantly correlated with daily internet use, suggesting that the association between academic progression and cyberchondria severity cannot be explained simply by longer daily internet exposure.

This study offers a comprehensive overview of the gender-related dynamics influencing both perceived e-health literacy and cyberchondria profiles among the sampled student population. Female participants had slightly higher eHEALS scores than male participants, although this difference was close to the conventional threshold of statistical significance and should therefore be interpreted cautiously. This pattern may be considered in relation to previous evidence suggesting gender-related differences in health information seeking, health awareness, and engagement with health-promoting behaviors [31,32]. Regarding susceptibility to cyberchondria, female university students scored significantly higher than male students. The observed gender disparity lends further empirical support to prior investigations indicating that the psychological burden of cyberchondria may be more pronounced among women than men [33]. Additionally, eHEALS scores differed across academic years, with fourth-year students showing the highest scores compared with students in lower years. This may reflect increasing academic exposure, familiarity with health-related content, and greater confidence in accessing and evaluating online health information as students progress through their education. This finding may be interpreted in light of previous evidence indicating that health literacy-related skills are important for evaluating online health information and judging its credibility [34]. Department-based differences were also observed for both eHEALS and CSS-12 scores. Coaching Education students had the highest scores for both outcomes, whereas Sports Management students had the lowest CSS-12 scores. These differences may reflect variation in curricular emphasis, professional orientation, or exposure to performance- and health-related topics across departments.

In higher education, developing personal e-health literacy skills is important not only for health professions but also for future sports professionals who need to be technologically literate and capable of critically evaluating online health information. To effectively verify and control the accuracy of digital medical data, students need to strengthen not only their access to online information but also their ability to appraise the credibility, relevance, and emotional impact of such information. For this purpose, integrating digital health literacy initiatives into the educational curricula of students who will become future sports professionals has become increasingly important. Previous studies have already examined the relationship between perceived e-health literacy and cyberchondria among undergraduate and health sciences students using similar scale-based approaches [13,18,19]. By focusing specifically on sports science students, this study extends previous work conducted in general university or health sciences populations and highlights the relevance of perceived e-health literacy and cyberchondria within a student group whose academic training is closely connected to physical health, performance, injury prevention, and rehabilitation. Therefore, this contribution should be interpreted as context-specific descriptive evidence rather than as evidence of a new theoretical mechanism or a fundamentally distinct student profile. Because no comparison group from other faculties was included, the present findings do not demonstrate that sports science students have a different digital health or cyberchondria profile compared with students from other academic disciplines. However, the present findings suggest that such interventions should not focus solely on improving access to online health information. They should also emphasize critical appraisal, source verification, misinformation awareness, and emotional self-regulation during health-related internet searches.

While this study provides important information, its cross-sectional design limits causal inference and underlines the need for future longitudinal research to validate these associations. Because the dataset was obtained from only one institutional setting, caution should be exercised in generalizing these results to different academic populations or institutional contexts. In addition, the study did not include a comparison group from other faculties; therefore, it cannot determine whether sports science students differ from students in other academic disciplines in terms of perceived e-health literacy or cyberchondria severity. The exclusion of 26 students because of incomplete questionnaire data represents another limitation. Since these incomplete forms did not consistently contain sufficient demographic and scale-related information, a formal comparison between included and excluded participants could not be performed; therefore, a small degree of selection bias cannot be completely excluded. In addition, although the regression model adjusted for age, gender, daily internet use, academic year, and department, potentially relevant psychological and clinical factors such as baseline health anxiety, previous anxiety symptoms, injury history, and performance-related anxiety were not assessed. Athlete status, sport-related symptom-searching behavior, and individual-level curricular exposure were also not measured, limiting the ability to interpret the observed associations as sports-specific psychological or behavioral mechanisms. These unmeasured variables may have influenced the observed associations. Therefore, the positive association between perceived e-health literacy and CSS-12 score should not be interpreted as independent of pre-existing health anxiety or other psychological vulnerability traits. Reverse directionality is also possible; students with greater cyberchondria severity may engage in more frequent online health searches, which may in turn be reflected in higher perceived e-health literacy scores. Future studies should utilize longitudinal and multicenter designs to better understand causal mechanisms and investigate mediating and moderating factors linking perceived e-health literacy and cyberchondria. In addition, comparative studies including students from different academic disciplines and sport-specific measurements are needed to determine whether sports science students have a distinct digital health or cyberchondria profile. The gathered evidence underscores that merely enhancing digital health literacy does not guarantee a concomitant reduction in cyberchondria severity among higher education cohorts. To improve health information literacy outcomes, health educators should integrate specific educational strategies into their pedagogical practices. These strategies include teaching students to develop critical evaluation skills when assessing information presented in digital media, manage emotional responses through self-regulation, and evaluate evidence-based health information to achieve more adaptive psychological outcomes. Overall, the integration of these strategies may help improve students’ health information literacy while reducing the potential risks associated with cyberchondria-related behaviors.

## 5. Conclusions

This study highlights the complex relationship between perceived e-health literacy and cyberchondria among undergraduate sports science students. Although participants demonstrated eHEALS scores close to the upper end of the possible scale range, they also exhibited considerable cyberchondria severity scores, suggesting that greater perceived competence in accessing and using online health information does not necessarily protect individuals from health-related anxiety or repetitive symptom-searching behaviors. The significant positive association between perceived e-health literacy and cyberchondria indicates that students who feel more confident in navigating digital health resources may also be more exposed to excessive, conflicting, or anxiety-provoking health information. This association remained statistically significant but weak after adjustment for daily internet use, gender, age, academic year, and department. Daily internet use was also associated with both constructs and emerged as one of the stronger predictors of cyberchondria severity, emphasizing the central role of digital engagement in contemporary health information-seeking practices. Gender, academic year, and department were also associated with cyberchondria severity, suggesting that sociodemographic and educational context should be considered when interpreting these patterns. These findings suggest that educational interventions should move beyond improving technical access to online health information and should instead focus on critical appraisal, misinformation recognition, responsible information-seeking behavior, and emotional self-regulation. By integrating these components into student-centered health education programs, universities may help students benefit from digital health resources while minimizing the psychological risks associated with excessive online health searching. Future multicenter and longitudinal studies are needed to clarify the direction of these relationships and to develop evidence-based strategies for promoting healthier engagement with digital health information.

## Figures and Tables

**Table 1:** Sociodemographic Parameters of the Sampled Population.

Variable	*n*	%
Female	220	53.4
Male	192	46.6
Sports Management	148	35.9
Coaching Education	136	33.0
Physical Education Teaching	128	31.1
First-year students	95	23.1
Second-year students	104	25.2
Third-year students	103	25.0
Fourth-year students	110	26.7

**Table 2:** Central Tendency and Dispersion Statistics for the Composite Scores.

Variable	*n*	Possible Range	Mean ± SD	Median	Observed Range
eHEALS	412	8–40	37.1 ± 7.4	40.0	8.0–40.0
CSS-12	412	12–60	31.8 ± 9.1	32	14–55
Daily Internet Use	412	Not applicable	4.9 ± 2.2	4.9	1.0–12.0

Values are presented as mean ± standard deviation unless otherwise stated. CSS-12 was analyzed as a continuous score on the original 12–60 metric. For consistency with conventional CSS-12 total-score reporting, the median and observed range are presented as whole-number descriptive values. This presentation did not affect the inferential analyses, regression coefficients, confidence intervals, *p*-values, or conclusions. eHEALS denotes the e-Health Literacy Scale; CSS-12 denotes the Short-Form Cyberchondria Severity Scale.

**Table 3:** Comparative Analysis of eHEALS and CSS-12 Scores Evaluated by Sociodemographic and Educational Variables.

Outcomes	Variable	Category	*n*	Mean ± SD/Overall Test
eHEALS	Gender	Female	220	37.8 ± 6.1
eHEALS	Gender	Male	192	36.4 ± 8.6
eHEALS		Overall test		Welch *t*-test: t(339.8) = 1.97, *p* = 0.050; d = 0.20 [95% CI 0.00 to 0.39]
eHEALS	Daily Internet Use	<3 h/day	85	29.9 ± 12.2
eHEALS	Daily Internet Use	3–6 h/day	205	38.5 ± 4.7
eHEALS	Daily Internet Use	>6 h/day	122	39.9 ± 0.6
eHEALS		Overall test		Welch ANOVA: F(2.0, 161.5) = 38.13, *p* ≤ 0.001; η^2^ = 0.26 [95% CI 0.17 to 0.35]
eHEALS	Department	Sports Management	148	35.0 ± 9.6
eHEALS	Department	Coaching Education	136	39.0 ± 4.0
eHEALS	Department	Physical Education Teaching	128	37.7 ± 6.7
eHEALS		Overall test		Welch ANOVA: F(2.0, 245.4) = 11.04, *p* ≤ 0.001; η^2^ = 0.05 [95% CI 0.02 to 0.10]
eHEALS	Academic Year	First year	95	33.5 ± 10.4
eHEALS	Academic Year	Second year	104	35.7 ± 8.7
eHEALS	Academic Year	Third year	103	39.3 ± 3.5
eHEALS	Academic Year	Fourth year	110	39.6 ± 2.9
eHEALS		Overall test		Welch ANOVA: F(3.0, 204.4) = 15.23, *p* ≤ 0.001; η^2^ = 0.11 [95% CI 0.07 to 0.17]
CSS-12	Gender	Female	220	33.2 ± 9.1
CSS-12	Gender	Male	192	30.2 ± 9.0
CSS-12		Overall test		Student *t*-test: t(410.0) = 3.28, *p* = 0.001; d = 0.32 [95% CI 0.13 to 0.52]
CSS-12	Daily Internet Use	<3 h/day	85	25.8 ± 8.4
CSS-12	Daily Internet Use	3–6 h/day	205	31.3 ± 8.0
CSS-12	Daily Internet Use	>6 h/day	122	36.9 ± 8.7
CSS-12		Overall test		ANOVA: F(2, 409) = 45.37, *p* ≤ 0.001; η^2^ = 0.18 [95% CI 0.12 to 0.25]
CSS-12	Department	Sports Management	148	25.2 ± 6.9
CSS-12	Department	Coaching Education	136	37.7 ± 7.6
CSS-12	Department	Physical Education Teaching	128	33.2 ± 8.1
CSS-12		Overall test		ANOVA: F(2, 409) = 101.90, *p* ≤ 0.001; η^2^ = 0.33 [95% CI 0.26 to 0.41]
CSS-12	Academic Year	First year	95	28.3 ± 7.5
CSS-12	Academic Year	Second year	104	29.6 ± 9.2
CSS-12	Academic Year	Third year	103	33.0 ± 8.3
CSS-12	Academic Year	Fourth year	110	35.7 ± 9.5
CSS-12		Overall test		ANOVA: F(3, 408) = 15.51, *p* ≤ 0.001; η^2^ = 0.10 [95% CI 0.06 to 0.17]

Values are presented as mean ± standard deviation. Overall test rows include the test statistics, degrees of freedom, *p*-value, effect size, and 95% confidence interval. eHEALS denotes the e-Health Literacy Scale; CSS-12 denotes the Short-Form Cyberchondria Severity Scale; CI denotes confidence interval.

**Table 4:** Correlations Between Perceived E-Health Literacy, Internet Use, Academic Characteristics, and Cyberchondria Severity.

Variable Pair	Method	*n*	Correlation Coefficient	95% CI	*p*-Value
Academic year—daily internet use	Spearman correlation	412	rho = 0.02	−0.07 to 0.12	0.647
Academic year—eHEALS score	Spearman correlation	412	rho = 0.47	0.40 to 0.55	<0.001
Academic year—CSS-12 score	Spearman correlation	412	rho = 0.31	0.22 to 0.40	<0.001
Daily internet use—eHEALS score	Pearson correlation	412	r = 0.45	0.37 to 0.52	<0.001
Daily internet use—CSS-12 score	Pearson correlation	412	r = 0.53	0.45 to 0.59	<0.001
eHEALS score—CSS-12 score	Pearson correlation	412	r = 0.54	0.46 to 0.60	<0.001

Pearson correlation was used for associations between continuous variables. Spearman correlation was used for analyses involving academic year because academic year is an ordinal variable. eHEALS denotes the e-Health Literacy Scale; CSS-12 denotes the Short-Form Cyberchondria Severity Scale; CI denotes confidence interval.

**Table 5:** Multivariable linear regression analysis for CSS-12 score.

Predictor	B	95% CI	Standardized Beta	*p*-Value	VIF
eHEALS score	0.07	0.00 to 0.14	0.06	0.041	1.27
Daily internet use, h/day	2.04	1.81 to 2.28	0.49	<0.001	1.15
Gender: female vs. male	3.85	2.96 to 4.74	0.21	<0.001	1.02
Age, years	−0.28	−0.58 to 0.02	−0.04	0.071	1.01
Academic year	2.70	2.28 to 3.12	0.33	<0.001	1.10
Department: Coaching Education vs. Sports Management	12.30	11.22 to 13.37	0.63	<0.001	1.02
Department: Physical Education Teaching vs. Sports Management	7.77	6.69 to 8.85	0.39	<0.001	1.02

Model summary: *n* = 412, R^2^ = 0.757, adjusted R^2^ = 0.753, F(7, 404) = 179.85, *p* < 0.001. Diagnostic checks: maximum VIF = 1.27; residual normality, Shapiro–Wilk W = 0.994, *p* = 0.115; heteroscedasticity, Breusch–Pagan BP = 15.42, *p* = 0.031; maximum Cook’s distance = 0.029. The dependent variable was CSS-12 score. B denotes the unstandardized regression coefficient. Confidence intervals and *p*-values were calculated using HC3 robust standard errors because the Breusch–Pagan test indicated heteroscedasticity. CI denotes confidence interval; VIF denotes variance inflation factor. eHEALS denotes the e-Health Literacy Scale; CSS-12 denotes the Short-Form Cyberchondria Severity Scale. Sports Management and male gender were used as reference categories.

## Data Availability

The dataset is not publicly available because it was obtained from human participants and includes survey-based demographic and educational information. Although the data used in the analyses were anonymized, unrestricted public sharing of the full dataset may still raise privacy and confidentiality concerns.

## Supplementary Materials

The following supporting information can be downloaded at: https://www.mdpi.com/article/10.3390/healthcare14172862/s1, Table S1. Post hoc pairwise comparisons for significant multi-group analyses.

## Abbreviations

CSS-12, Short-Form Cyberchondria Severity Scale; eHEALS, e-Health Literacy Scale; H1, Hypothesis 1; H2, Hypothesis 2; SD, standard deviation; SPSS, Statistical Package for the Social Sciences.
