# Supplementary material for: Perceived E-Health Literacy and Cyberchondria Among Undergraduate Sports Science Students: A Cross-Sectional Study
Source: Healthcare (Basel). 2026 Sep 5;14(17):2862. doi: 10.3390/healthcare14172862 (PMC13565353; doi:10.3390/healthcare14172862)
Supplement: Supplementary file 1 [file healthcare-14-02862-s001.zip › healthcare-4454967-supplementary.pdf]

Supplementary Table S1. Post hoc pairwise comparisons for significant multi-group analyses.

Variable	Outcome	Method	Comparison	Mean difference	95% CI	Adjusted p-value
Daily Internet Use	eHEALS	Pairwise Welch t-test with Holm correction	<3 hours/day vs 3–6 hours/day	-8.60	-11.29 to -5.90	<0.001
Daily Internet Use	eHEALS	Pairwise Welch t-test with Holm correction	<3 hours/day vs >6 hours/day	-10.04	-12.67 to -7.42	<0.001
Daily Internet Use	eHEALS	Pairwise Welch t-test with Holm correction	3–6 hours/day vs >6 hours/day	-1.44	-2.10 to -0.79	<0.001
Daily Internet Use	CSS-12	Tukey HSD	3–6 hours/day vs <3 hours/day	5.44	2.92 to 7.95	<0.001
Daily Internet Use	CSS-12	Tukey HSD	>6 hours/day vs <3 hours/day	11.06	8.30 to 13.82	<0.001
Daily Internet Use	CSS-12	Tukey HSD	>6 hours/day vs 3–6 hours/day	5.62	3.39 to 7.86	<0.001
Department	eHEALS	Pairwise Welch t-test with Holm correction	Sports Management vs Coaching Education	-3.98	-5.69 to -2.28	<0.001
Department	eHEALS	Pairwise Welch t-test with Holm correction	Sports Management vs Physical Education Teaching	-2.71	-4.65 to -0.76	0.013
Department	eHEALS	Pairwise Welch t-test with Holm correction	Coaching Education vs Physical Education Teaching	1.27	-0.07 to 2.62	0.063
Department	CSS-12	Tukey HSD	Coaching Education vs Sports Management	12.50	10.41 to 14.59	<0.001
Department	CSS-12	Tukey HSD	Physical Education Teaching vs Sports Management	8.02	5.90 to 10.15	<0.001
Department	CSS-12	Tukey HSD	Physical Education Teaching vs Coaching Education	-4.48	-6.65 to -2.31	<0.001
Academic Year	eHEALS	Pairwise Welch t-test with Holm correction	First year vs Second year	-2.20	-4.90 to 0.50	0.219
Academic Year	eHEALS	Pairwise Welch t-test with Holm correction	First year vs Third year	-5.78	-8.01 to -3.55	<0.001
Academic Year	eHEALS	Pairwise Welch t-test with Holm correction	First year vs Fourth year	-6.04	-8.23 to -3.85	<0.001
Academic Year	eHEALS	Pairwise Welch t-test with Holm correction	Second year vs Third year	-3.58	-5.39 to -1.77	<0.001
Academic Year	eHEALS	Pairwise Welch t-test with Holm correction	Second year vs Fourth year	-3.84	-5.60 to -2.08	<0.001
Academic Year	eHEALS	Pairwise Welch t-test with Holm correction	Third year vs Fourth year	-0.26	-1.13 to 0.60	0.550
Academic Year	CSS-12	Tukey HSD	Second year vs First year	1.34	-1.84 to 4.53	0.697
Academic Year	CSS-12	Tukey HSD	Third year vs First year	4.74	1.54 to 7.93	<0.001
Academic Year	CSS-12	Tukey HSD	Fourth year vs First year	7.45	4.31 to 10.60	<0.001
Academic Year	CSS-12	Tukey HSD	Third year vs Second year	3.39	0.27 to 6.51	0.027
Academic Year	CSS-12	Tukey HSD	Fourth year vs Second year	6.11	3.04 to 9.18	<0.001
Academic Year	CSS-12	Tukey HSD	Fourth year vs Third year	2.72	-0.36 to 5.79	0.105

Post hoc comparisons were performed only when the global multi-group comparison was statistically significant. Tukey HSD was used when the homogeneity of variance assumption was acceptable; otherwise, pairwise Welch t-tests with Holm correction were applied.
